# Artificial Lighting for Public Institutions

**Published:** 1898-12-03

**Authors:** Carlton Lambert

**Affiliations:** Royal Naval College, Greenwich.


### THE INSTITUTIONAL WORKSHOP.

### ARTIFICIAL LIGHTING FOR PUBLIC INSTITUTIONS.

### GAS AND ELECTRIC.

By Professor Carlton Lambert, M.A., Royal Naval College, Greenwich.

(*Continued from page* 153.)

As some provincial institutions have no access to public electric mains, the following estimate may be useful. It represents the cost of laying down an electric generating plant and installation of 300 16-candle lamps, and the annua outlay for the maintenance of the light, together with the cost of an equivalent of gas lighting.

Electricity:—Outlay on Plant—

| | |
|---|---|
| Gas Engine, 35 brake horse-power ... ... ... | £300 |
| Dynamo, 25 kilo-watts ... ... ... ... ... | 160 |
| Accumulators ... ... ... ... ... ... | 350 |
| Lamps, Switches, Wire, Fitting, &c. ... ... ... | 275 |
| | £1,085 |

Annual Expenditure, 1,500 hours—

| | |
|---|---|
| Renewal of Lamps, 600, at 1s. 6d. ... ... ... | £45 |
| Depreciation and up-keep, 10 per cent. on £810 ... | 81 |
| Labour ... ... ... ... ... ... ... | 40 |
| Gas and Oil ... ... ... ... ... ... ... | 110 |
| Interest on Plant, 4 per cent. on £810 ... ... ... | 32 |
| | £308 |

Gas:—Welsbach Incandescent: Annual Cost for Equal Light—

| | |
|---|---|
| Gas at 2s. 6d. per 1,000 ft. ... ... ... ... | £65 |
| Renewals of Mantles, &c. ... ... ... ... ... | 17 |
| | £82 |

If current had been supplied from electric mains at 6d. per Board of Trade unit, the annual cost of electric lighting, including lamp renewals, would have been ... ... ... ... ... ... £765

The one main and undoubted advantage offered by electric lighting is that it does not pollute the air we have to breathe. In the old days of gas lighting this air pollution was in ill-ventilated rooms, a more serious matter, but now that we get the same light as formerly by burning only from one-sixth to one-tenth the amount of gas, this objection to gas lighting has practically vanished.

A little calculation will make this clear: A room containing 3,000 cubic feet is amply lighted by a single Welsbach. If the ventilation is as usually provided, the circulation through the flue and other outlets and inlets will cause the air contents of the room to be thoroughly changed at least three to four times per hour, the circulation being, in fact, assisted by a gas burner through the differences of temperature which it sets up. Thus we may say that about 10,000 cubic feet of outside air enter the room hourly. Now a Welsbach burner, consuming 3·6 cubic feet of gas per hour, produces about 2 cubic feet of carbonic acid gas in the same time. After the lamp is lighted the extra quantity of carbonic acid in the room, resulting from the gas, will gradually, in an hour or so, rise to its maximum, and can never exceed 2 per 10,000 of air. Now this is about half of what already exists in the mountain air of Ben Nevis, and may be altogether disregarded in its effect on health.

Of course, when it is said that because an adult expires only ·6 cubic feet of carbonic acid hourly, therefore a gas-burner is as bad as three or four adults, this is only true in the matter of the comparatively innocuous carbonic acid gas which they put forth. It is the organic exhalations from human beings which are a real source of danger, and not the products of combustion of a good gas-burner.

The writer in 1894 made a careful test of the purity of combustion in the Welsbach burners. Not the slightest trace of acetylene could be detected, and it was a fair inference that no carbon-monoxide was present. Subsequent direct tests by a committee appointed by the *Lancet* established conclusively the entire absence of carbon-monoxide. Even the competitors of gas lighting do not now venture to suggest that the combustion in a Welsbach burner is otherwise than perfect. The flame in the new Welsbach burner is, perhaps, the most perfect Bunsen flame ever produced, the inner cone being reduced to a beautiful blue ring not more than three millimetres high, indicating an almost theoretically perfect mixture of gas and air.

It has often been remarked that the Welsbach light is trying to the eyes. Of course this is true of any powerful light if looked at directly, but lights are not made to be looked at, they are intended to illuminate objects which we want to look at. The eyes are usually and easily protected from the direct light of a lamp by opal or other screens.

Oculists do not appear to have put forth any quantitative statements on this subject, but it seems natural to assume that the tiring effect on the eye would depend on one or both of the two following factors: (1) The total amount of luminous energy reaching the retina, and (2) the intrinsic intensity of the image of a bright body. If the first factor is the main one, then diffused daylight should be far more injurious than many Welsbach lamps. If the latter, then a glowing electric filament would be more likely to produce local injury to the retina of the eye on account of the smallness of the image in which the light is concentrated compared with that of a Welsbach mantle at the same distance.

To ensure the best success in the every-day use of Welsbach lamps, attention to a few simple details is necessary, and it may be useful to enumerate here the chief instructions given to those who manage the lights at the Yarrow Home, and which have conduced to the satisfactory results obtained there:—

1. Don't leave the gas tap full on, but turn it back until the best light is shown. The gas flame should just fill the mantle. Any more gas burnt gives less light and is only wasted.
2. If a black deposit of carbon should form at the top of the mantle, this is either due to a dirty burner or too much gas being burned. Turn the gas down till the flame only fills one-half of the mantle, giving very little light; the deposit will, in a few minutes, disappear by oxydation.
3. When lighting a burner at the top of the chimney, wait a few seconds after turning the gas on and before applying the light; this prevents shock to the mantle.
4. Burners, especially in dusty rooms, sometimes get clogged by dirt and give poor light. When this is the case, carefully remove the mantle from the rod; take off the gallery, and blow vigorously through the gauze or scrub it with a brush. Remove any dust also from the gas nipple by blowing through the air-holes at the bottom of the burner; replace and re-light. This almost invariably results in a great increase in the light.

The following extract from the report, just received, of the Richmond Public Library Committee shows the result of the removal of the "Wenham" gas burners in that institution and their substitution by "incandescent" burners, in the effecting of which the writer was instrumental. It concludes this article, and should encourage those who are meditating the installation of Welsbach lights:—

"The adoption of the incandescent system of gas lighting referred to in last year's report has quite satisfied all expectations as regards increased cleanliness, superior quality of the light, greater purity of the atmosphere, and reduction in the amount of gas consumed. The saving in expense by the new system during the year 1897-8 amounted to £21 13s. 4d., or 32 per cent. of the previous year's cost."

